# Association Between Atherosclerotic Cardiovascular Disease (ASCVD) Risk Score and Arteriovenous Fistula Failure in Patients on Maintenance Hemodialysis

**DOI:** 10.7759/cureus.62298

**Published:** 2024-06-13

**Authors:** Gajashree M, Sandhya Suresh, Appan Prakash, Geethanjali G, Ram Prasad Elumalai, Manikantan Shekar, Jayakumar M

**Affiliations:** 1 Nephrology, Sri Ramachandra Institute of Higher Education and Research, Chennai, IND

**Keywords:** mineral and bone disorder, chronic kidney disease, atherosclerotic cardiovascular disease, hemodialysis, vascular access failure

## Abstract

Background: Arteriovenous fistula (AVF) is the vascular access of choice for hemodialysis in end-stage renal disease (ESRD) patients but has a significant failure rate. Atherosclerotic cardiovascular disease (ASCVD) is a major cause of mortality in ESRD patients. Atherosclerosis of the peripheral vessels may contribute to poor maturation of AVF leading to the exploration of the ASCVD score as a prognostic tool for AVF failure.

Methods: This study included 110 hemodialysis patients with AVFs and aimed to examine the association between ASCVD score and AVF failure. Participants were categorized into the presence of vascular access failure (N=12) and absence of vascular access failure (N=98), and demographic and clinical data were collected.

Results: The study comprised predominantly male patients (63.6%), with a notable prevalence of hypertension and diabetes. Twelve patients experienced AVF failure, with pseudoaneurysms and thrombosis being the predominant causes. The ASCVD risk group at intermediate and high stages exhibited a statistically significant risk (relative risk (RR)=1.403; 95% CI, 1.041-1.904) of AVF failure in comparison to the low and borderline ASCVD risk groups. There was no association of age, gender (male and female), body mass index (BMI), serum calcium, serum phosphorus, intact parathyroid hormone (iPTH), and serum albumin with AVF failure.

Conclusion: The ASCVD score emerges as a potential prognostic tool to identify dialysis patients at high risk of AVF failure, suggesting avenues for targeted interventions and improved patient care. However, limitations of the ASCVD risk estimator and study limitations, such as small sample size and absence of mortality data, warrant cautious interpretation and necessitate further exploration in larger patient populations.

## Introduction

Chronic kidney disease (CKD) poses a significant global health burden, affecting millions of individuals and demanding continuous medical intervention to mitigate its complications [1]. Among the therapeutic modalities, maintenance hemodialysis stands as a mainstay in the management of end-stage renal disease (ESRD) [2]. However, the efficacy of hemodialysis critically depends on the maintenance of reliable vascular access, which serves as the lifeline for effective blood filtration during each dialysis session [3]. The vascular access options commonly employed in hemodialysis include arteriovenous fistulas (AVFs), arteriovenous grafts (AVGs), and central venous catheters (CVCs) [4]. While AVFs are considered the gold standard due to their superior longevity and lower complication rates, the incidence of permanent vascular failure remains a formidable challenge with a study from India showing a one-year patency of 62.6% [5]. Cardiovascular disease (CVD) stands as the primary cause of mortality among individuals undergoing chronic hemodialysis [6,7]. Cardiovascular alterations arising from renal dysfunction, encompassing factors such as fluid overload, uremic cardiomyopathy, CKD-mineral and bone disorder (MBD), anemia, modified lipid metabolism, the buildup of uremic toxins derived from gut microbiota (e.g., trimethylamine N-oxidase), inflammation, oxidative stress, and various elements of the uremic environment, collectively heighten the susceptibility to CVD within the dialysis population [8,9]. As CVD continues to exert a formidable impact on the morbidity and mortality of hemodialysis patients, the atherosclerotic cardiovascular disease (ASCVD) score has emerged as a widely accepted tool for assessing the risk of cardiovascular events [10,11]. Gender, age, smoking, and the presence of various comorbidities such as diabetes, cardiovascular issues, obesity, and frailty contribute to the failure of vascular access [12,13]. Overall, impaired vascular access diminishes the quality of life and negatively impacts patient-centered outcomes [14].

While the ASCVD score is well-established in predicting cardiovascular events, its potential as a prognostic tool for permanent vascular access complications has yet to be comprehensively explored. Therefore, this study was done to examine the relationship between the ASCVD score and the risk of permanent vascular access failure and determine the role of the ASCVD score as a predictor for AVF failure.

## Materials and methods

The study was conducted at Sri Ramachandra Institute of Higher Education and Research (SRIHER), Chennai, over a one-year period between March 2022 and February 2023 after getting approval from the Institute Ethical Committee (CSP/22/FEB/105/49). ESRD patients in our dialysis center were enrolled prospectively in the study at the completion of one year after the creation of the AV fistula. Patients who were below the age of 18 years and those on peritoneal dialysis were excluded. Details including patient demographics and biochemical parameters were collected and the ASCVD risk scores were also calculated according to the ASCVD risk estimator provided by the American Heart Association. All the study participants were divided into two groups based on the presence or absence of AVF failure, which was defined as an AV access that never matured adequately to be used successfully for dialysis or failure of an AV access in the one-year period since the start of hemodialysis.

Statistical analysis

The study characteristics were expressed as mean values ± standard deviations. All patients were divided into two groups depending on the presence or absence of AVF failure. The independent samples t-test was used to find the significance of the incidence of failure in relation to continuous variables. All statistical tests were two-tailed and P<0.05 was considered significant. The relative risk of AVF failure was determined for known risk factors including patients with high and intermediate ASCVD scores. The analysis was performed using the SPSS software, version 16.0 (SPSS, Chicago, IL, USA).

## Results

Patient characteristics

A total of 110 patients were included in the study. Among them, 52 patients (47.3%) were between 40 and 60 years of age while 43 patients (39.1%) were over 60 years of age. 53 patients (48.2%) had diabetes mellitus (DM) and 32 patients (29.1%) had a history of ischemic heart disease (IHD) (Table 1). Vascular access failure was present in 12 patients (10.9%). Among them, four were due to pseudo aneurysm formation, three due to thrombosis, two each with infections and stenosis, and one due to fistula infiltration. 22.7% of the patients belonged to the low ASCVD risk group and 20.9% belonged to the high-risk group (Table 1).

**Table 1:** Baseline characteristics The frequency has been represented as number (N) (%). IHD, ischemic heart disease; ASCVD, atherosclerotic cardiovascular disease

Baseline characteristics	N (%)
Age	
20-40 years	15 (13.6%)
40-60 years	52 (47.3 %)
60-90 years	43 (39.1 %)
Male	70 (63.6 %)
Female	40 (36.4 %)
Comorbid conditions	
Hypertension	110 (100%)
IHD	32 (29.1%)
DM	53 (48.2%)
Frequency of hemodialysis	
Twice weekly	78 (70.9%)
Thrice weekly	32 (29.1%)
History of vascular access failure present	12 (10.9%)
MBD present	19 (17.3%)
Albumin (g/dL)	
>3.5	57 (51.8%)
2.5-3.5	44 (40%)
<2.5	9 (8.2%)
ASCVD score	
Low risk	25 (22.7%)
Borderline risk	19 (17.3%)
Intermediate risk	43 (39.1%)
High risk	23 (20.9%)

Factors associated with vascular access failure

Age, gender, body mass index (BMI), and daily urine output did not have any significant relationship with vascular access failure. Serum calcium, phosphorus, and intact parathyroid hormone (iPTH) levels were comparable in the two groups. Patients with access failure tended to have lower serum albumin compared to patients without access failure (3.37±0.35 vs. 3.68±0.58, p=0.075) (Table 2).

**Table 2:** Correlation of continuous variables with history of vascular access failure The data has been represented as mean ± standard deviation. BMI, body mass index; iPTH, intact parathyroid hormone

Variable	Vascular access failure present (n=12)	No vascular access failure (n=98)	P-value
Age	61.5±12.8	55.11±13.4	0.122
Male	9 (75%)	60 (61.22 %)	0.529
BMI	22.98±1.99	24.74±4.37	0.172
Urine output	212.50±253.74	290.82±288.97	0.372
Calcium	9.05±1.33	8.54±0.83	0.067
Phosphorous	4.71±2.00	4.55±1.59	0.745
iPTH	256.23±-216.01	296.51±264.93	0.614
Albumin	3.37±0.35	3.68±0.58	0.075

Association of ASCVD risk with vascular access failure

**Figure 1:**
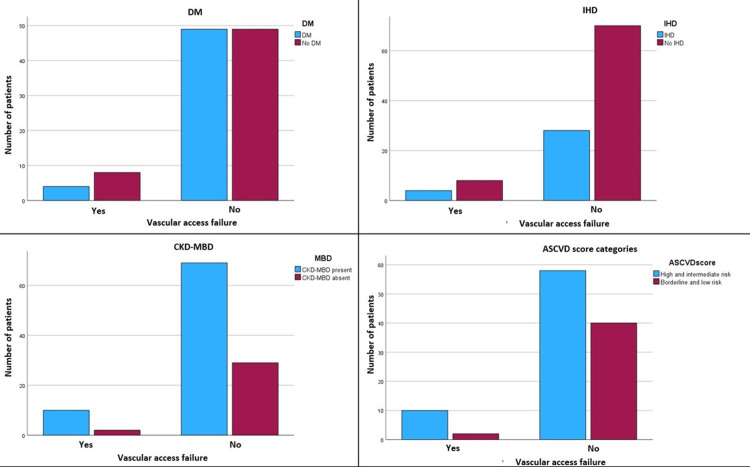
Distribution of variables in patients with and without a history of vascular access failure The proportion of patients with AVF failure in patients with and without DM (1a) and IHD (1b), MBD (1c) and among those in low- (<7.5%) and high-risk (≥7.5%) ASCVD categories ASCVD, atherosclerotic cardiovascular disease; DM, diabetes mellitus; IHD, ischemic heart disease; AVF, arteriovenous fistula; CKD-MBD, chronic kidney disease-mineral and bone disorder

There was no significant association between DM, IHD, and MBD and AVF failure (Figure 1). The relative risk of vascular access failure was significantly higher in the intermediate and high ASCVD risk group compared to the low and borderline ASCVD risk group (relative risk=1.408, 95% CI=1.041-1.904) (Table 3).

**Table 3:** Relative risk of vascular access failure associated with risk factors The data for vascular access failure present and vascular access failure absent is represented as number (N) (%) while risk ratio is represented as RR (lower limit of 95% CI-upper limit of 95% CI). DM, diabetes mellitus; IHD, ischemic heart disease; CKD-MBD, chronic kidney disease-mineral and bone disorder; ASCVD, atherosclerotic cardiovascular disease

Risk factors	Vascular access failure present (N=12)	No vascular access failure (N=98)	RR (95% CI)
DM	4 (33.3%)	49 (50%)	0.667 (0.292-1.520)
IHD	4 (33.3%)	28 (28.6%)	1.167 (0.494-2.755)
CKD-MBD	10 (83.3%)	69 (70.4%)	1.184 (0.891-1.572)
ASCVD high and intermediate risk score	10 (83.3%)	58 (59.2%)	1.408 (1.041-1.904)

## Discussion

This retrospective study aimed to shed light on the complex interplay between cardiovascular health, calculated through the ASCVD score, and the risk of AVF failure in hemodialysis patients. Studies by Wanner et al. have highlighted a strong association between ventricular hypertrophy, hypertension, and type 2 DM in individuals undergoing dialysis [15]. Additionally, these studies have revealed that cardiac and vascular mortality rates were significantly elevated among dialysis patients compared to the general population. In our study, there was a significant proportion of the population with risk factors for ASCVD with all patients having hypertension and nearly half the cohort having diabetes.

AVF is considered the gold standard for hemodialysis; however, AVF failure may occur in as many as 20-60% of patients [5,16]. In our study, we found that 12 out of 110 patients developed AVF failure in the first year after creation, the causes of which included pseudoaneurysms, fistula infiltration, thrombosis, stenosis, and infections. There are diverse challenges in maintaining functional vascular access and it is important to determine the predictive factors for vascular access failure, which would enable the institution of preventive measures. Atherosclerosis and arteriosclerosis affecting the arteries of the upper limbs can adversely affect AVF maturation and have also been shown to be associated with cardiovascular events [17]. Hence, we aimed to study the association of ASCVD risk factors as determined by the ASCVD risk estimator with the likelihood of AVF failure.

The ASCVD scoring algorithm calculates the 10-year risk of having a CVD and uses parameters such as age, sex, presence of diabetes, history of smoking, race, and serum cholesterol levels to calculate the cardiovascular risk profile. It stratifies patients into low-risk, borderline-risk, intermediate-risk, and high-risk categories [11]. The findings from this study revealed a statistically significant increase in the risk of vascular access failure in the intermediate and high ASCVD risk groups. This highlights the potential utility of the ASCVD score as a prognostic tool specifically in predicting vascular access complications, a novel aspect that extends the traditional use of this score in assessing cardiovascular events. However, it should be noted that the ASCVD risk equation was developed from a pooled cohort to estimate the 10-year risk of the first ASCVD event and it was not validated in the CKD population [11,18]. This estimator has several limitations as it does not include other factors contributing to ASCVD in hemodialysis patients such as disorders of mineral and bone metabolism, endothelial dysfunction, and inflammation, which limits its use in this population. It also does not take into account dialysis-related factors such as intradialytic hypertension, hypotension, and net ultrafiltration. Modifications of this cardiovascular risk prediction model are being tested in dialysis patients but the ASCVD risk score from the pooled cohort equation can be easily estimated from online calculators from patient's available data [19]. While the sample size for our study was small and there are studies determining the association between cardiovascular mortality and AVF patency, this is one of the first studies to evaluate the association between the ASCVD risk scoring by the pooled cohort equation and AVF failure. Further studies evaluating modifications of this equation in predicting AVF failure are required.

Additionally, the study aimed to determine the association of AVF failure with other known risk factors. Advancing age has been identified as a cause of poor AVF maturation and survival, although quantifying this association proves challenging due to age also serving as a surrogate marker for an increasing burden of comorbidities. A retrospective study suggested that the rate of AV fistula failure increased by 1% after the age of 67 years [20,21]. Research also indicates that outcomes of AV fistulae are notably poorer in females compared to males, though the precise reasons for this discrepancy remain unclear [22,23]. While it has been suggested that females may have smaller vessels with associated reduced luminal diameters compared to males, this factor has not consistently been identified as a cause of unsuccessful AVFs [24,25]. However, our study showed no relationship between age, sex, and vascular failure contrary to the above studies. Outflow dynamics are subject to various influencing factors, and one such factor is obesity. In our study, there was no significant association between BMI and vascular access failure. Obesity is identified as a risk factor for the failure of vascular access, distinct from the heightened incidence of diabetes within this population. A study by Kats et al. noted that obese patients exhibited poor secondary patency, with the underlying theory attributing this outcome to increased soft tissue mass causing venous compression and obstruction in the outflow tract [26,27]. Other variables such as serum calcium, serum phosphorus, iPTH, and serum albumin were examined, and the study did not identify statistically significant associations with the presence or absence of vascular access failure. This suggests the complexity of factors influencing access outcomes and emphasizes the need for a comprehensive understanding of individual patient profiles.

The identification of ASCVD risk as a predictor of vascular access failure opens avenues for targeted interventions in hemodialysis patients. Monitoring ASCVD risk may aid in the early identification of individuals at higher risk of access complications, allowing for pre-emptive measures and tailored management strategies. The limitations of the study, such as a small sample size, lack of patient follow-up, absence of cardiac evaluation, and no inclusion of mortality data, warrant careful interpretation. It is also important to note the limitations of the ASCVD risk equation in the hemodialysis population. The utility of modified cardiovascular risk prediction models that take into account specific risk factors in the dialysis population to predict AVF failure must be determined by further studies.

## Conclusions

This study contributes to the evolving understanding of the interplay between cardiovascular risk and vascular access outcomes in hemodialysis patients. The ASCVD score may emerge as a potential prognostic tool for assessing the baseline risk of AVF failure, urging further exploration and validation in larger and diverse patient populations. Further investigation with the addition of risk factors specific to the dialysis population such as mineral-bone disease, markers of inflammation and endothelial dysfunction, and intradialytic events is required.
